# Developmental changes of opsin gene expression in ray-finned fishes (Actinopterygii)

**DOI:** 10.1098/rspb.2022.1855

**Published:** 2022-11-09

**Authors:** Nik Lupše, Monika Kłodawska, Veronika Truhlářová, Prokop Košátko, Vojtěch Kašpar, Arnold Roger Bitja Nyom, Zuzana Musilova

**Affiliations:** ^1^ Department of Zoology, Faculty of Science, Charles University, Vinicna 7, 12844 Prague, Czech Republic; ^2^ Faculty of Fisheries and Protection of Waters, South Bohemian Research Center of Aquaculture and Biodiversity of Hydrocenoses, Research Institute of Fish Culture and Hydrobiology, University of South Bohemia in České Budějovice, Zátiší 728/II, 389 25 Vodňany, Czech Republic; ^3^ Department of Management of Fisheries and Aquatic Ecosystems, University of Douala, Douala P.O. Box 7236, Cameroon; ^4^ Department of Biological Sciences, University of Ngaoundéré, Ngaoundéré P.O. Box 454, Cameroon

**Keywords:** opsin, evolution, Actinopterygii, development, vision, gene expression

## Abstract

Fish often change their habitat and trophic preferences during development. Dramatic functional differences between embryos, larvae, juveniles and adults also concern sensory systems, including vision. Here, we focus on the photoreceptors (rod and cone cells) in the retina and their gene expression profiles during development. Using comparative transcriptomics on 63 species, belonging to 23 actinopterygian orders, we report general developmental patterns of opsin expression, mostly suggesting an increased importance of the rod opsin (*RH1*) gene and the long-wavelength-sensitive cone opsin, and a decreasing importance of the shorter wavelength-sensitive cone opsin throughout development. Furthermore, we investigate in detail ontogenetic changes in 14 selected species (from Polypteriformes, Acipenseriformes, Cypriniformes, Aulopiformes and Cichliformes), and we report examples of expanded cone opsin repertoires, cone opsin switches (mostly within *RH2*) and increasing rod : cone ratio as evidenced by the opsin and phototransduction cascade genes. Our findings provide molecular support for developmental stage-specific visual palettes of ray-finned fishes and shifts between, which most likely arose in response to ecological, behavioural and physiological factors.

## 1. Introduction

Fish visual systems are very diverse, and they vary in morphology, physiology and spectral sensitivity [1–3]. Vertebrate vision is enabled by cone and rod photoreceptors in the retina, which carry light-sensitive molecules composed of an opsin protein bound to a light absorbing, vitamin A-derived chromophore [4]. In fishes, there are usually four types of cone opsins (*SWS1* and *SWS2;* commonly found in single cones, whereas *RH2* and *LWS* in double cones; with the respective peak sensitivity ranges of 347–383 nm, 397–482 nm, 452–537 nm and 501–573 nm; [2]) used for photopic and colour vision, and one rod opsin (rhodopsin, *RH1* or Rho) for scotopic vision in dim-light conditions [2]. Through gene duplications followed by functional diversifications, extant teleost fishes reached a median of seven cone opsin genes within their genomes [5]. Throughout the phylogeny, teleost genomes contain more copies of double-cone genes (middle and longer wavelength sensitive; *RH2* and *LWS*) than that of single cones (shorter wavelength *SWS1* and *SWS2*). While the *SWS1* is often missing from the genome or seen in one, at best two copies [3] and *SWS2* seen in up to three copies [6], teleost genomes can contain up to eight copies of *RH2* [7] and up to five copies of *LWS* [8]. Unlike cone opsins, rod opsin duplicates are rarely found, most often in mesopelagic lineages [5,9,10]. Higher copy number is considered beneficial by providing more ‘substrate’ for selection, as well as for alternative gene expression of the variants within the opsin type.

The formation of the eye, and expression of opsin genes, starts at the embryonic stage [11,12]. Still, eyes continue to grow, and new photoreceptors are being added throughout life [13]. Within the retina, cone photoreceptors are first to develop, followed by temporally and spatially distinct rods [14–16]. For example, in zebrafish, photoreceptor progenitor cells start out by first differentiating into cones before rods are added later during development [17], suggesting that vision changes with age. This cone-to-rod developmental sequence is likely shared across vertebrates (Atlantic cod: [18]; zebrafish: [17]; mice: [19]; rhesus monkey: [20] and appears to hold even for teleost species with an all-rod retina in the adult stage [10]).

Photic conditions can change spatially and temporally, resulting in a visually heterogeneous environment in which visual systems of fishes are expected to be under natural selection that favours those that match the local environment [21]. For example, longer and shorter wavelengths are scattered and filtered out with increasing water depth and consequently, fishes living in deep-water habitats such as sculpins of Lake Baikal [22], cichlids of lakes Malawi and Tanganyika [23,24], and African crater lakes [25,26], as well as deep-sea fishes [10,27] have visual systems sensitive to the blue-green part of the visible spectrum. Adaptation can be achieved either through functional diversification of opsin genes when mutations at key-spectral tuning sites shift the peak spectral sensitivity (*λ*_max_) of the photopigment [28,29], or by regulation of the opsin gene expression. This can be achieved when a subset of opsin genes is expressed and altered among or within species and even within the same individuals during ontogeny [10,21,30,31].

Before reaching the juvenile or sexually mature adult stage, fish larvae undergo major anatomical, physiological, behavioural and quite often, ecological changes [2,32]. The developmental shift in habitat preference is often suggested to drive ontogenetic changes in opsin expression (e.g. cichlids: [21,33]; black bream: [34]; eel: [35]; squirrelfishes and soldierfishes: [36]; clown anemonefish: [37]; damselfishes: [38]; bluefin killifish: [39]; gambusia: [40]; rainbow trout: [41]; dottybacks: [42]; starry flounder: [43]; deep-sea fishes: [10,44]). However, habitat-related changes of photic conditions solely do not always result in different and stage-specific visual system modifications, as seen in the Atlantic cod [18] or the spotted unicornfish [45]. Shifts in diet (planktivory, carnivory and herbivory) and activity patterns (diurnal, nocturnal and crepuscular) [36,46,47], in addition to developmental or phylogenetic constraints seem to also play a role in shaping the visual diversity of fishes and potential age-related shifts of it.

Here, we aim to investigate ontogenetic changes of opsin and phototransduction cascade gene expression across ray-finned fishes, to estimate the presence and relative abundance of opsin gene classes, and to elucidate general and/or taxon-specific patterns. For the purpose of this study, we have sequenced and analysed (i) retinal transcriptomes of different developmental stages of 14 species, belonging to five major actinopterygian orders: *Polypterus senegalensis* (Polypteriformes), *Acipenser ruthenus* (Acipenseriformes), *Abramis brama* and *Vimba vimba* (both Cypriniformes), *Scopelarchus* spp. and *Coccorella atlantica* (both Aulopiformes), *Coptodon bemini*, *C. imbriferna*, *C. flava*, *C. snyderae*, *C. thysi*, *Sarotherodon linnellii*, *S. lohbergeri* and *Stomatepia pindu* (all Cichliformes from the Bermin and Barombi Mbo lakes). (ii) We have complemented this dataset by publicly available embryonic/larval/juvenile/adult transcriptomes belonging to 49 species and 21 orders, some of which have never been analysed for visual gene expression before. In total, the comprehensive dataset of 63 species from 23 ray-finned fish orders allows us to focus on the development of the opsin gene expression, and rod and cone cell identity throughout actinopterygian evolution.

## 2. Methods and materials

### (a) Data and sample collection

**Figure 3.**
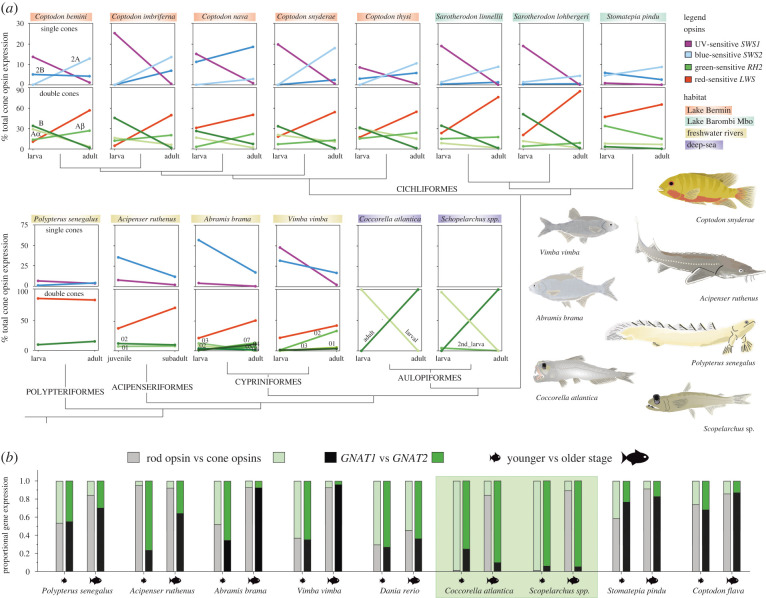
Cone opsin gene switches, age-specific copies and phototransduction cascade gene expression of representative taxa specifically sequenced for this study. (*a*) Detailed presentation of ontogenetic changes of opsin expression in selected polypteriform, acipenseriform, cypriniform, aulopiform and cichliform species. Interconnected dots are coloured according to specific single- and double-cone opsins and present mean expression values for specific developmental stages. In cases of gene duplications, copies are named and coloured with different shades. *Y*-axis scale, which is identical for all species depicted in the same row but differs between Cichliformes and all other fish orders, is labelled only on the left most axis. For details on number of individuals and exact values, see electronic supplementary table. (*b*) Ontogenetic changes of rod/cone opsin gene expression, and to it related shifts in expression of phototransduction cascade genes GNAT1 (rod specific) and GNAT2 (cone specific) for selected teleost taxa. Highlighted in green are special cases of the two aulopiform species that exhibit a discordance between the dominating opsin type (rod specific) and phototransduction cascade genes (cone specific) in adults [10]. (Online version in colour.)

Transcriptomes belonging to taxa deemed as focal groups, which were inspected for age-specific copies and presented in detail in figure 3, were obtained from specimens (*N* = 73) caught solely for the purpose of this study. In detail, 16 specimens were classified as larvae, 4 as juveniles, 3 as subadults and 50 as adults (figure 3; electronic supplementary material, table). *Polypterus senegalensis* larvae were collected in the rearing facility of the Department of Zoology, Charles University, and the adults were purchased from the aquarium trade. *Acipenser ruthenus* and cyprinids were collected at the rearing facility in Vodňany, and in local water bodies (adults: Velky Tisy pond, Klicava dam, Lipno dam; larvae: Vltava and Elbe rivers), Czech Republic, respectively. Both mesopelagic taxa, *Scopelarchus* spp. and *Coccorella atlantica*, were collected in the Sargasso Sea and originate from Lupše *et al*. [10]. Crater lake cichlids were collected in lakes Barombi Mbo and Bermin (Cameroon, West Africa) between 2013 and 2018 (research permit numbers: 0000047,49/MINRESI/B00/C00/C10/nye, 0000116,117/MINRESI/ B00/C00/C10/C14, 000002-3/MINRESI/B00/C00/C10/C11, 0000032,48-50/MINRESI/B00/C00/C10/C12). Larvae were caught by fine-meshed nets and fixed in RNAlaterTM immediately. Adults were collected using gill nets and selective capturing by snorkelling in the shallow-water zone. For all species, fin clips were taken from specimens and stored in 96% EtOH for subsequent molecular analyses. Larval samples were fixed in RNAlaterTM (ThermoFisher) and stored at −80°C until further use. Adults of all species were euthanized on site with eyes or retinae extracted, fixed in RNAlaterTM and stored at −80°C upon arrival at the laboratory.

**Figure 1.**
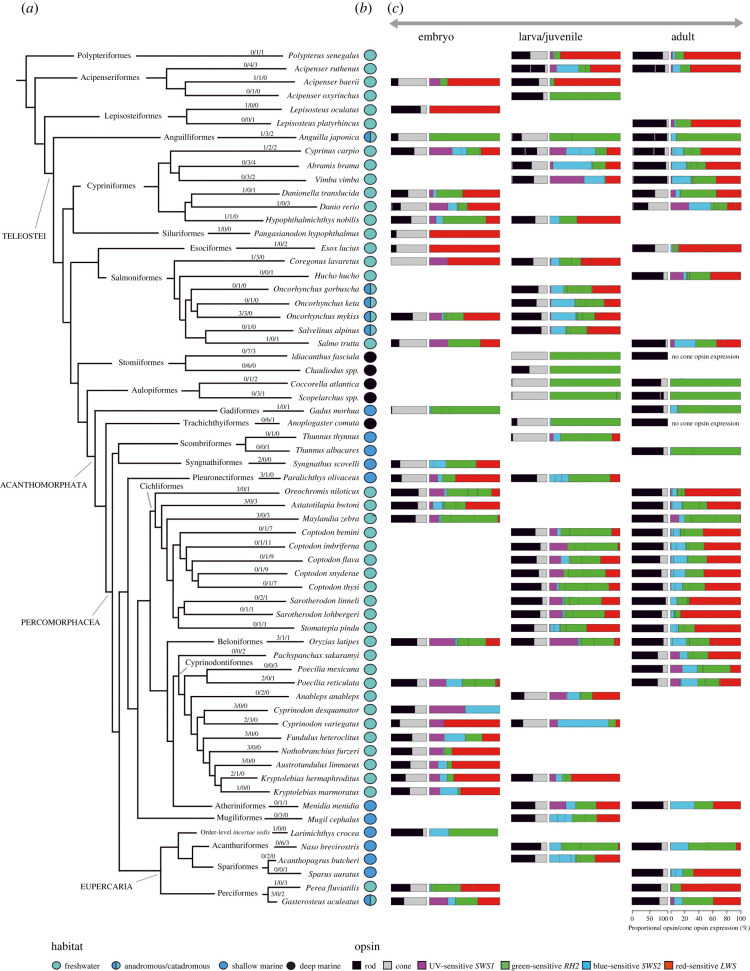
Opsin gene expression in different developmental stages of ray-finned fishes (Actinopterygii). (*a*) Simplified phylogeny of the 63 species, belonging to 23 orders, for which the transcriptomes were analysed (topology after [48]). Numbers above branches represent number of individuals per developmental stage analysed (embryo/larva + juvenile/adult). (*b*) Information on habitat preference, obtained from https://www.fishbase.de. Separation between the shallow and deep marine species is 200 m. Information on depth obtained from https://obis.org/. (*c*) Proportional opsin gene expression (horizontal bars) at different developmental stages. First (shorter) bar represents mean proportional expression of rod and cone opsins. Cone opsin expression (grey) is depicted as the sum of the expression of all four classes of cone opsin genes (*SWS1*, *SWS2*, *RH2* and *LWS*). If several rod opsin genes (black) were expressed, the different proportions of their expression are distinguished with white vertical bars. Second (longer) bar represents mean proportional expression of different cone opsins. Black vertical bars within gene classes separate different copies, if co-expressed. For details, see electronic supplementary material, table. (Online version in colour.)

To obtain publicly available transcriptomes used in this study (figure 1; electronic supplementary material, table), we searched the largest publicly available repository of high-throughput sequencing data, the Sequence Read Archive (SRA), using the following topic search term: ‘(embryo* OR larva* OR juvenile* OR adult*) AND (retina* OR eye* OR head* OR whole*) AND (taxon name * OR fish*)’. Whenever possible, we have analysed up to three specimens per stage per species (figure 1; electronic supplementary material, table). In the case of embryos, specimens closest to hatching were analysed. The entire dataset analysed, including de novo transcriptomes described below, includes 215 samples of which, based on morphology, 56 were classified as embryos, 40 as larvae, 25 as juveniles, 3 as subadults and 91 as adults (figures 1 and 3; electronic supplementary material, table). Sample IDs, number of raw reads, individual accession numbers for BioProject PRJNA841439 and further parameters are listed in the electronic supplementary material, table.

### (b) Transcriptome sequencing and analyses

Total RNA was extracted from the whole eyes or retinal tissue using either the RNeasy micro- or mini-kit (Qiagen). The extracted RNA concentration and integrity were verified on a 2100 Bioanalyzer (Agilent) and Qubit Fluorometer (Thermofisher Scientific). RNA-seq libraries were constructed in-house from unfragmented total RNA using Illumina's NEBNext Ultra II Directional RNA library preparation kit, NEBNext Multiplex Oligos and the NEBNext Poly(A) mRNA Magnetic Isolation Module (New England Biolabs). Multiplexed libraries were sequenced on the Illumina HiSeq 2500 platform as 150 bp paired-end reads. The sequence data was quality checked using FastQC [49]. Opsin gene expression was then quantified using Geneious software version 11.0.3 [50]. For each sample, we mapped the reads against a general genomic reference dataset for all visual opsin genes composed of Nile tilapia, zebrafish and the long-nose gar, using the Medium-sensitivity settings in Geneious. This enabled us to capture most of the cone and rod opsin-specific reads and create species-specific opsin references. If needed, paralogous genes were subsequently disentangled following the methods in Musilova *et al*. [5] and de Busserolles *et al*. [51]. Transcriptome reads were then re-mapped to the newly created (species-specific) references with medium-low sensitivity to obtain copy-specific expression levels. We report opsin gene proportional expression in relation to the total opsin gene expression which was calculated using FPKM (Fragments Per Kilobase of transcript Per Million reads), taking into account the library size, the length of each gene and number of mapped reads (electronic supplementary material, table). The above-mentioned quantification of opsin gene expression was also used on transcriptomes obtained from SRA. Identical pipeline was used for the quantification of *GNAT1*/*2* genes in selected taxa (figure 3).

### (c) Statistical analyses

To formally test whether opsin gene expression differs between developmental stages, we applied the beta regression models specifically designed to analyse the proportional datasets and percentages. We used the R package betareg [52], which allows handling of non-transformed data. The beta distribution has a highly flexible shape and is, hence, suitable to fit the dependent variable (in our case the proportional expression of each opsin gene) in the standard unit interval (0,1) with a mean related to a set of categorical regressors (in our case developmental stage). We tested the difference for each cone opsin gene class separately (i.e. *SWS1*, *SWS2*, *RH2* and *LWS*), then for the sum of single-cone (*SWS1* + *SWS2*), and double-cone opsins (*RH2* + *LWS*), and additionally also for rods (*RH1*) and cones (*SWS1* + *SWS2* + *RH2* + *LWS*).

## 3. Results and discussion

### (a) General developmental patterns of opsin gene expression across the actinopterygian phylogeny—cone-to-rod developmental constraint

**Figure 2.**
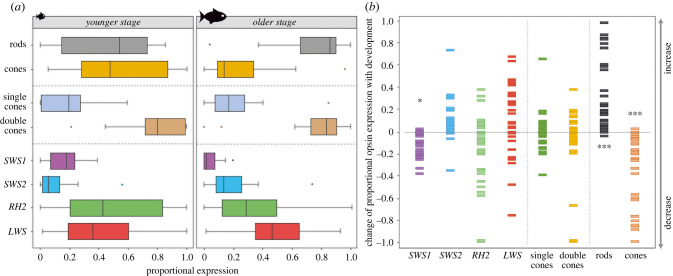
General patterns of age-related opsin expression changes. (*a*) Interquartile ranges (25th and 75th percentiles) and whiskers show data dispersion (proportional expression) across different opsins for the youngest and oldest analysed stage. Data medians are presented as solid vertical lines. To avoid over-representation of certain taxa (e.g. five Coptodon species), data points (*N* = 32) represent mean genus values and are comprised only of species that had at least two developmental stages analysed. (*b*) Change of opsin expression (positive/negative) with development, calculated as a difference between the mean opsin expression in the oldest and the youngest stage of a certain genus. Resulting values are represented by rectangles (*N* = 32), centred at the mean. The lower half of the plot (values below 0.0) shows a decrease, and the upper half (values above 0.0) an increase in proportional expression with age. Significant differences found by beta regression models are marked by asterisks (table 1 for details). (Online version in colour.)

The analysis of the opsin gene expression in 63 ray-finned fishes revealed that, generally, the ratio of the rod opsin (*RH1* or Rho, *λ*_max_: 447–525 nm) to cone opsin expression increases with age in analysed species (figures 1 and 2, table 1; electronic supplementary material, table; *p* ≤ 0.001). This is in accord with the cone-to-rod development of the retina which starts with cone cells, and rods appearing only later [10,17,18]. The increasing rod : cone cell ratio is further confirmed by the expression of the phototransduction cascade gene *GNAT1* (rod specific) versus *GNAT2* (cone specific), figure 3*b*. Rod opsin and *GNAT1* usage increases significantly already during the larval and juvenile stage, before finally transforming into sexually mature adults with rod-dominant retina (figures 1 and 2; electronic supplementary material, table). It thus seems that larval vision is mostly driven by cone vision, while the ability to perform well in low-light conditions appears consequently, at later developmental stages [32,53]. Functionally, rods generally allow for an improvement in visual acuity and startle responses in fishes [54–56] and are also associated with motion sensitivity and the appearance of novel behaviours, such as schooling [57]. More specifically, higher rod expression increases individual performance of fishes living in the deep-sea [10,51]. Additionally, laboratory experiments have shown that the ability to follow a rotating stripe pattern (the optomotor drum) might be dependent on rod formation and retinal development, as it is not seen in stages or specimens lacking rods [58–60].

**Table 1.** Statistical comparison between the younger and older developmental stages for 32 ray-finned fish genera. Summary of beta regression models specifically aimed at proportional datasets (opsin expression as a dependent variable from developmental stage) with the obtained *p*-values. Alpha levels of significance after the Bonferroni correction additionally marked as equivalent to: <0.001*** and <0.05*.

opsin gene(s)	*p*-value
*SWS1*	0.005*
*SWS2*	0.040
*RH2*	0.469
*LWS*	0.675
rods	1.6 × 10^−9^***
cones	7.6 × 10^−10^***
single cones	0.950
double cones	0.302

In the selected taxa (figure 3), we have specifically focused on the rod versus cone identity by quantifying the expression of the phototransduction cascade gene *GNAT1* or *GNAT2*, respectively. We found correspondence between the expression of phototransduction cascade gene type and the opsin type (i.e. cone *SWS1*, *SWS2*, *RH2*, *LWS* and rod *RH1*), and detected a clear increase of *GNAT1* : *GNAT2* ratio with ageing, with the exception of the Aulopiformes deep-sea fishes. In this group, a discordance between the dominating opsin type (rod specific) and phototransduction cascade genes (cone specific) in adults challenges the rod versus cone identity and suggests a presence of possibly partially transmuted photoreceptors, potentially similar but not identical to other vertebrates (snakes and geckoes: [61,62]; deep-sea fishes: [10,51,63]; salamanders: [64]). The overall intriguing visual system of aulopiforms, hence, definitely needs to be investigated further and in more detail (figure 3, [10]).

### (b) Developmental switch of the short-wavelength-sensitive opsin genes

A trend of age-related shifts in expression also appears within cone opsins (table 1). Our dataset shows a clear decrease in proportional expression of the UV-sensitive *SWS1* (*λ*_max_: 347–383 nm) with age (*p* = 0.005; table 1). Although *SWS1* expression is usually low, it seems to be expressed more in early stages throughout the phylogeny (figure 1, table 1). On one hand, UV radiation can result in larval mortality; to mitigate negative effects of exposure, UV avoidance through detection of UV light and adjustments of vertical position is expected [65,66]. On the other hand, distinguishing wavelengths belonging to the UV part of the visual spectrum aids younger individuals that feed on zooplankton [67–69]. With ageing and a shift in diet, UV opsin expression might become irrelevant for some species [70], thus potentially explaining why some adults do not express *SWS1* (e.g. *Naso brevirostris* and *Oryzias latipes*), while others still do (e.g. *Danio rerio*, *Poecilia reticulata* and cichlids) (figure 1; electronic supplementary material, table). Adult expression of *SWS1*, when seen, seems to play a role in species and/or colour discrimination and mate selection (guppies: [71]; damselfishes: [72]; cichlids: [21]), male aggression (sticklebacks: [73]) or is associated with migration events (salmonids: [41]). The blue-sensitive *SWS2* cone opsin (*λ*_max_: 397–482 nm) expression generally increases with age and generally replaces the *SWS1* gene in the single cones (figures 1 and 2; electronic supplementary material, table, table 1). Interestingly, while some fish (e.g. sturgeons and cyprinids) seem to ontogenetically decrease the proportion of both *SWS1* and *SWS2* opsins, other fish groups (e.g. cichlids) replace one type by another (figure 3). This switch in single-cone opsin expression between *SWS1* and *SWS2* has been shown before, e.g. by Spady *et al*. [74] in Nile tilapia or by Cheng and Flamarique [75] in rainbow trout, and it mostly keeps the total single-cone opsin expression similar between different developmental stages (figure 2).

### (c) Middle- and long-wavelength-sensitive opsins in double cones

The ontogenetic switch in expression also occurs between the green-sensitive *RH2* (*λ*_max_: 452–537 nm) and the red-sensitive *LWS* (*λ*_max_: 501–573 nm) cone opsin types; plus switching between different *RH2* copies also commonly occurs (figure 3). Values for these typically double-cone opsins vary considerably across the fish phylogeny, albeit a possible weak general trend of a decrease in relative expression of *RH2*, and an increase of *LWS* with age is noticable (figures 1 and 2; electronic supplementary material, table; not significant—table 1), except for groups that completely lost the *LWS* opsin gene. In general, medium-wavelength opsins appear to be of use to all stages (figures 1 and 2; electronic supplementary material, table), perhaps due to the general presence of corresponding wavelengths in most habitats. Our overview data further seem to show that freshwater species exhibit dominance of red-sensitive *LWS* opsin gene expression, whereas in marine species, green-sensitive *RH2* gets to be more dominant (with exceptions) (figure 1). Namely, for species inhabiting the spectrally narrower deep sea at least during certain parts of their lives (Stomiiformes, Aulopiformes, Trachichthyiformes, Anguilliformes and Gadiformes), *RH2* seems to be the most important, if not the only cone opsin expressed (figure 1, [10]). On the other hand, the expression of *LWS* in adults might be a response to inhabiting freshwater habitats, such as turbid rivers and murky, eutrophic lakes (e.g. Lake Victoria) where usually, longer wavelengths penetrate to greater depths and are the most prevalent colour of the ambient light [21,76]. The expression of *LWS* is also beneficial for foraging in herbivorous reef fishes, providing them with the visual ability to discriminate benthic algae from coral reef backgrounds [77,78]. In some cases, increased *LWS* expression and expanded *LWS* repertoires might also be explained by sexual selection (e.g. in Poeciliidae), where females evolved mate preferences for red and orange male colouration [79].

### (d) Age-specific cone opsin gene copies in the selected taxa

We have specifically focused and de novo sequenced retina transcriptomes of larvae/juveniles and adults of 14 actinopterygian species belonging to five orders spanning the ray-finned fish phylogeny. Apart from the aforementioned rod versus cone identity assessed by *GNAT* genes, we have additionally focused on switches between copies of the same opsin type in the selected taxa (figure 3; electronic supplementary material, table). Namely, we studied the visual opsin gene repertoire in two basal non-teleost fish groups, bichirs (Polypteriformes) and sturgeons (Acipenseriformes), and in teleost riverine cyprinids (Cypriniformes; Ostariophysi), crater lake cichlids (Cichliformes; Euteleostei) and deep-sea pearleyes and sabretooths (Aulopiformes; Euteleostei). The overall expression patterns are in most cases in accord with the general patterns discussed above (figure 3; electronic supplementary material, table), with exceptions seen in the deep-sea fishes (based on our earlier data from [10]).

In all species but the bichir, we found multiple copies within at least one opsin gene type, namely within the rod *RH1* opsin, and cone *SWS2* and *RH2* opsins. In some species (cyprinids, sturgeon, *Scopelarchus* spp.) we found simultaneous expression of two rod *RH1* copies (figure 1; electronic supplementary material, table). All three groups possess the two *RH1* genes in their genome resulting from three independent ancestral gene duplication events [3,10]. The *RH1* gene duplicates were lost in the later evolution of the euteleost crown group, and hence most teleost species carry only one *RH1* copy, a phenomenon similar to that seen in ‘non-fish’ vertebrates. These *RH1* copies do not show any sign of ontogenetic switch in studied species, as known, e.g. for eels [80]. On the other hand, we detected several cases of stage-specific copies within cone opsin genes. While *Acipenser ruthenus* and *Abramis brama* *+* *Vimba vimba* express only one *SWS2* copy, cichlids express two different *SWS2* genes (figure 3; electronic supplementary material, table); this corresponds to multiple copies found in their genome due to the neoteleost- and percomorph-specific *SWS2* gene duplications [6]. Most examined species show an expanded *RH2* repertoire (figure 3; electronic supplementary material, table) and the existence of clearly larval and adult-specific copies has been observed in cyprinids, cichlids and in the deep-sea aulopiforms (figure 3). The expression of multiple copies might enhance colour vision by increased spectral resolution useful in a particular environment; however, reasons for these opsin switches are not yet completely understood. The presence of such stage-specific copies means that species adjust their vision to differing light environments not only through a change in opsin class expression, but also through preferential expression of opsin copies within a single class. In cichlids, a group for which the development of the visual system is probably best understood, a shift to longer wavelength copies is generally observed within a single opsin type (*RH2A* copies replacing *RH2B* with age) or among single-cone opsins (*SWS2* replacing *SWS1*) and has been reported before for different groups of cichlids (e.g. Malawi, [12]; Nile tilapia, [74]).

Mesopelagic deep-sea aulopiform species have a limited repertoire of cone opsin classes that reflects living in photon-depleted depths [10,26]. *Scopelarchus* spp. and *Coccorella atlantica* express only one cone opsin class, namely *RH2* (figure 3; electronic supplementary material, table). However, both expanded their *RH2* repertoires and express larval- and adult-specific copies that are thought to be functionally different and most likely best respond to different wavelengths shallow-water epipelagic larvae and mesopelagic deep-water adults encounter (figure 3; electronic supplementary material, table) [10]. Genomic analyses by Lupše *et al*. [10] reveal a total of three, and seven *RH2* cone opsin copies within the genomes of *Coccorella atlantica* and *Scopelarchus michaelsarsi*, respectively. Mesopelagic fish lineages in some cases expand rod opsin repertoires, which are better suited for dim-light conditions [10,26]. *Coccorella* and *Scopelarchus*, however, seem to inhabit relatively shallower and photon-richer depths than some other deep-sea fishes, such as Stomiiformes, and might thus benefit also from having extra copies of cone opsins [10].

We have collected a robust dataset combining not only our own, but also publicly available genetic data, deposited in databases. This allowed us to detect shared versus specific expression patterns among different fish groups. We are aware that the collected dataset has certain limitations and that many factors could not be controlled in this study. For example, this dataset is highly dependent on publicly available material, so there is no control over several potentially relevant factors, such as the sampling conditions, intraspecific variability, other tissues sequenced together with the eyes (as in the entire embryos), etc. Since not all stages are available for all species, we do not present any typical ‘developmental time series’ but rather snapshots of embryos, larvae, juveniles and adults; consequently, more subtle or time-restricted expression patterns could not be detected here. For the purpose of statistical analyses, we have restricted the public dataset only to species with two (or more) stages found (figure 2). To complement the public data we also include our own, controlled data in more detail (figure 3). Despite certain limitations, our combined dataset provides robust evidence for expression patterns shared across distantly related fish groups, as it highlights general trends, and more detailed conclusions achieved through in-detail analyses of species specifically sequenced within this study.

## 4. Conclusion

To conclude, this study aimed to identify general patterns of the visual opsin gene expression shared among ray-finned fishes, and to detect similarities in the ontogenetic changes between opsin gene types. We found that the rod : cone opsins ratio increased with age in fish species, supporting the conserved cone-to-rod developmental pathway. We also report the increasing importance of the *LWS*, and the decreasing importance of the *SWS1* opsin genes with age, observed across ray-finned fish phylogeny (e.g. in sturgeons, cyprinids and cichlids). We have further detected the existence of different stage-specific *RH2* copies, which are switched during development. To conclude, fish visual systems are evolutionary and developmentally very dynamic and future studies focused on particular fish groups promise to throw further light on exact mechanisms, patterns and reasons for this extreme sensory system diversity.

## Data Availability

The raw Illumina reads from RNA-seq of all studied individuals are deposited into the NCBI BioProject database with ID PRJNA841439. Individual accession numbers are listed in the electronic supplementary material, table. The data are provided in the electronic supplementary material [81].
